# Transcriptome Analysis of a Premature Leaf Senescence Mutant of Common Wheat (*Triticum aestivum* L.)

**DOI:** 10.3390/ijms19030782

**Published:** 2018-03-10

**Authors:** Qiang Zhang, Chuan Xia, Lichao Zhang, Chunhao Dong, Xu Liu, Xiuying Kong

**Affiliations:** Key Laboratory of Crop Gene Resources and Germplasm Enhancement, Ministry of Agriculture, The National Key Facility for Crop Gene Resources and Genetic Improvement, Institute of Crop Science, Chinese Academy of Agricultural Sciences, Beijing 100081, China; zhangqiang9024@126.com (Q.Z.); xiachuan@caas.cn (C.X.); zhanglichao@caas.cn (L.Z.); dongchunhao1222@163.com (C.D.)

**Keywords:** wheat, leaf senescence, mutant, RNA-seq, differentially expressed genes

## Abstract

Leaf senescence is an important agronomic trait that affects both crop yield and quality. In this study, we characterized a premature leaf senescence mutant of wheat (*Triticum aestivum* L.) obtained by ethylmethane sulfonate (EMS) mutagenesis, named *m68*. Genetic analysis showed that the leaf senescence phenotype of *m68* is controlled by a single recessive nuclear gene. We compared the transcriptome of wheat leaves between the wild type (WT) and the *m68* mutant at four time points. Differentially expressed gene (DEG) analysis revealed many genes that were closely related to senescence genes. Gene Ontology (GO) enrichment analysis suggested that transcription factors and protein transport genes might function in the beginning of leaf senescence, while genes that were associated with chlorophyll and carbon metabolism might function in the later stage. Kyoto Encyclopedia of Genes and Genomes (KEGG) pathway analysis showed that the genes that are involved in plant hormone signal transduction were significantly enriched. Through expression pattern clustering of DEGs, we identified 1012 genes that were induced during senescence, and we found that the *WRKY* family and zinc finger transcription factors might be more important than other transcription factors in the early stage of leaf senescence. These results will not only support further gene cloning and functional analysis of *m68*, but also facilitate the study of leaf senescence in wheat.

## 1. Introduction

Senescence plays a key role in the adaptability of plants. Effective senescence can enhance the adaptation of plants to the environment [1]. In crops, leaf senescence is an important agronomic trait that not only affects crop yield, but also affects crop quality [2,3]. Leaf senescence is the last stage of leaf development that can induce leaf cell death and nutrient transfer from senescing leaves to seeds or storage organs. This process is not a negative or unregulated degeneration step; rather, it is a positive step that involves fine gene regulation [4]. During senescence, the metabolism of leaf cells changes. Specifically, assimilation decreases the while catabolism is enhanced, e.g., chloroplast degradation occurs, the photosynthetic capacity decreases, and macromolecular material degrades [5]. Leaf senescence is affected by both internal and external factors. The former mainly include leaf age, foliar reactive oxygen species (ROS) levels, hormones, and sugar content. The latter mainly include lighting conditions, soil moisture, nutrient content, temperature, and pathogens [6,7]. Because the regulation of leaf senescence is so important and complicated, many studies on senescence-associated genes (SAGs) have been carried out in different plant species [5,8].

In recent years, with biotechnological development, many SAGs have been studied in various plant species. Many SAGs have been found to be involved in chlorophyll metabolism. For instance, *GmSARK*, which belongs to the LRR receptor-like kinase family, regulates leaf senescence by regulating chloroplast development and chlorophyll accumulation [9]. In *Arabidopsis thaliana*, *At4G25080*, *At5G54190*, *At1G44446*, and *At3G51820*, which encode magnesium protoporphyrin IX methyltransferase, protochlorophyllide reductase, chlorophyllide-a oxygenase, and bacteriochlorophyll a synthase, respectively, can affect chlorophyll synthesis and leaf development [10,11,12,13]. Another *A. thaliana* gene, pheophorbide a oxygenase (*PAO*), regulates chlorophyll degradation and leaf senescence [14]. Pentatricopeptide repeat (*PPR*) genes play important roles in regulating the expression of chloroplast genes, which results in leaf senescence in rice [15]. The *PPR53* gene in maize can also control the expression of chloroplast genes by regulating the stability and translation of specific chloroplast RNAs [16]. Many GATA-zinc finger transcription factors have been found to be involved in the chlorophyll metabolism pathway. Mutation of the *GNC* gene, which belongs to the GATA-zinc transcription factor family, can destroy the chlorophyll synthesis process in *A. thaliana* [17]. In soybean, the over-expression of *GmGATA44* can rescue the phenotype of the *A. thaliana gnc* mutant by reversing a deficiency in the chlorophyll content [18]. Overexpression of the GATA-zinc transcription factor *OsGATA12* can delay senescence and enhance rice yield by elevating the chlorophyll content of leaves [19]. Additionally, Cytokinin responsive GATA transcription factor 1 (*Cga1*) has been reported to influence plant architecture by regulating the chlorophyll content in rice [20].

In addition, genes involved in plant hormone signal transduction and stress resistance also play important roles in the regulation of leaf senescence. For instance, the genes that participate in the ethylene (ET), jasmonic acid (JA), abscisic acid (ABA), and salicylic acid (SA) pathways can positively regulate leaf senescence, while other genes that are involved in the cytokinin (CK), indoleacetic acid (IAA), and gibberellin (GA) pathways may negatively regulate leaf senescence [21,22,23,24]. Orthologous genes *OsNAP*, *GPC-B1* and *AtNAP*, belonging to the *NAC* transcription factor family, regulate leaf senescence through the ABA pathway, and promote the transcription of SAGs [25,26,27]. Receptor-like protein kinase 1 (RPK1), an ABA-inducible receptor kinase, positively regulates leaf senescence in *A. thaliana* [28]. *AtMYB44* is involved in stress resistance and leaf senescence through interactions with ABA receptor pyrabactin resistance 8 (*PYL8*) [29]. In addition, *AtWRKY53*, *AtWRKY54*, *AtWRKY70*, and *AtCAMs*, which are induced during leaf senescence and cell death, are involved in the SA signaling pathway [30,31]. As a CK receptor, *AHK3* transfers signals to downstream genes *AHPs* and *ARR2*, which can inhibit leaf senescence in *A. thaliana* [32]. IAA plays complicated roles in the regulation of leaf senescence. Studies have shown that the exogenous application of IAA could delay leaf senescence, and other reports have indicated that the early auxin response gene *small auxin-up RNA* (*SAUR)* could positively regulate leaf senescence [33,34]. *PYL9* and *OsSPL32* are involved in the stress response. Overexpression of *PYL9* can enhance drought resistance and accelerate leaf senescence [35], and mutation of *OsSPL32* can enhance the defense response and induce leaf senescence in rice [36].

Although wheat is one of the most important crops in the world and approximately 20 percent of global calories consumed originate from wheat (www.fao.org/faostat), we still know very little about leaf senescence in wheat. Studies have shown that premature senescence affects the yield and quality of wheat [2]. A comparison of early and normally senescing near-isogenic wheat lines suggested that the enzyme activities of antioxidant systems, such as superoxide (O^2−^) dismutase (SOD), catalase (CAT), ascorbate peroxidase (APX), and glutathione reductase (GR) were significantly inhibited. The destruction of the oxidation-reduction system is accompanied by an increase in the superoxide (O^2−^) and hydrogen peroxide (H_2_O_2_) contents, which may lead to early leaf senescence [8]. A study of the wheat stay-green mutant *tasg1* indicated that wheat leaf senescence was related to CK-regulated sucrose metabolism [15]. *Lr34*/*Yr18*/*Pm38* encodes an ATP-binding cassette (ABC) transporter that provides durable resistance to multiple fungal pathogens and induces flag leaf tip necrosis-like leaf senescence [37]. *GPC-B1* is an NAC family transcription factor that accelerates leaf senescence and affects the protein, zinc, and iron contents of wild wheat grains [27]. *TaNAMs* were down-regulated by RNAi technology, which led to the stay-green phenotype and the reduction in nutritive material transport from flag leaves to seeds [38]. The difficulty that is associated with wheat gene studies is caused by the complexity of the huge wheat genome, with a high ratio of repeat sequences [39]. Recently, in the wake of the development of sequencing and assembly technologies, the wheat reference genome has been greatly improved to provide a favorable condition for the study of wheat genes.

Using RNA-seq to study leaf senescence in wheat has rarely been reported. In the present study, RNA-seq analysis was performed with the leaf senescence mutant *m68* and the wild-type (WT) variety YZ4110 at different time points. Differentially expressed genes (DEGs) were identified between *m68* and the WT, and the Gene Ontology (GO) enrichment, and the Kyoto Encyclopedia of Genes and Genomes (KEGG) pathways of DEGs were analyzed at different time points. Analysis of transcriptome data will be helpful for understanding leaf senescence in wheat.

## 2. Results

### 2.1. Phenotypic Characterization and Genetic Analysis

**Figure 1:**
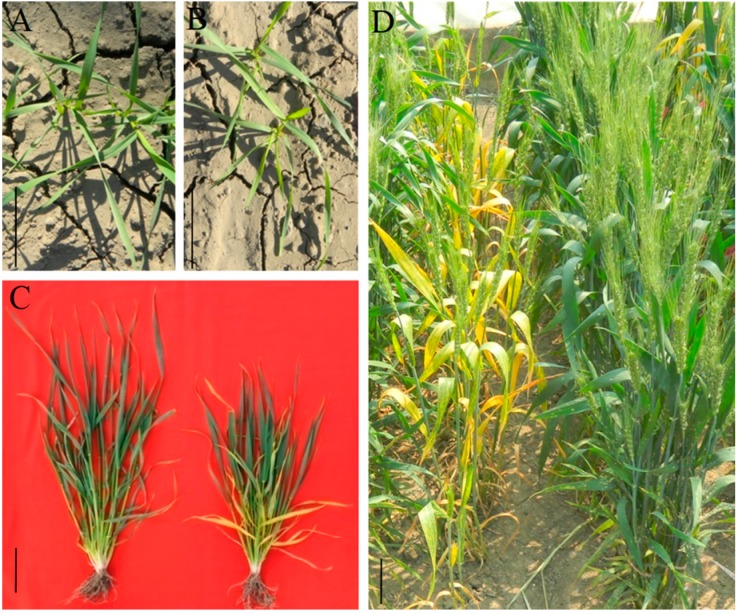
Phenotypic differences between the WT and *m68*. (**A**) The wild type (WT) phenotype at the seeding stage (**B**) The *m68* mutant phenotype at the seeding stage. (**C**) Leaf phenotypes of the WT (left) and *m68* mutant (right) at the shooting stage. (**D**) Filling stage phenotype of the *m68* mutant (left) and WT (right). Scale bars = 5 cm.

At the seedling stage, there was no obvious difference between the WT and *m68* mutant (Figure 1A,B). Initial senescence only occurred in the bottom leaves at the shooting stage in *m68* (Figure 1C). Then, the leaves in the middle part of *m68* plants showed senescence symptoms around the heading date. Approximately one week later, flag leaves started to senescence. During the filling stage, the leaf senescence process of the *m68* mutant accelerated dramatically (Figure 1D). The final spikelet number, grain number, floret number per spike, 1000-grain weight, plant height, and effective tiller number of *m68* were significantly different from those of the WT (Table 1). To determine the genetic basis of these phenotypes, we conducted genetic analysis. Among a total of 318 F_2_ plants, 73 showed the premature leaf senescence phenotype of *m68*, whereas the others showed the WT phenotype. The segregation ratio of the *m68* phenotype to the WT phenotype was 1:3.36. Based on the chi-square (χ^2^) test, the F_2_ population segregation ratio was in accordance with the expected ratio of 1:3 (χ^2^ = 0.71 < χ^2^_0.05,1_ = 3.84). The results indicated that premature leaf senescence of *m68* is controlled by a single recessive nuclear gene.

**Figure 2:**
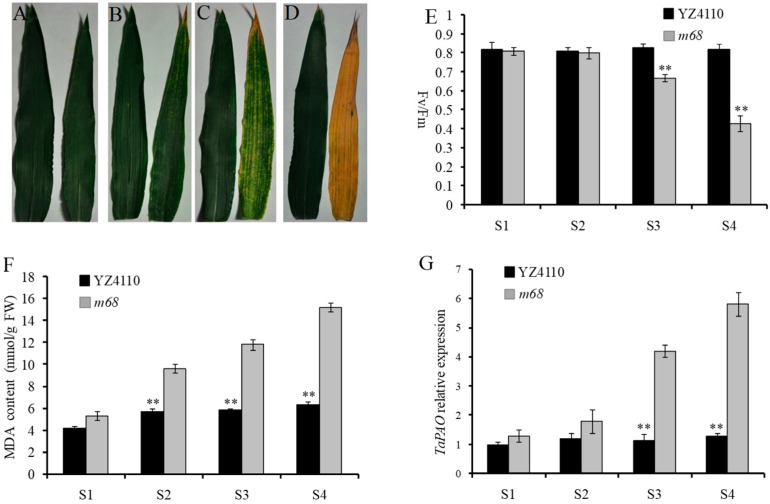
Phenotypic, physiological index measurements and *TaPAO* relative gene expression analysis of sample leaves of *m68* and the WT. (**A**–**D**) Leaves of the WT (left) and *m68* (right) were collected in S1 (**A**), S2 (**B**), S3 (**C**) and S4 (**D**). S1, S2, S3 and S4 represent stage 1 (19 April), stage 2 (25 April), stage 3 (3 May) and stage 4 (9 May), respectively; (**E**) Measurements of F_v_/F_m_ conducted on the WT and *m68* at different stages; (**F**) Measurements of the malondialdehyde (MDA) content of the WT and *m68* at different stages; (**G**) Relative expression of the *TaPAO* gene in the WT and *m68* at different stages. *GAPDH* was selected as the internal standard. Error bars represent the means ± SD (*n* = 3). ** indicates *p* < 0.01; * indicates *p* < 0.05.

As the development of flag leaves is crucial to agronomic traits, we observed the flag leaf phenotype of the *m68* mutant in detail beginning on the heading date, i.e., 19 April 2016. We separated the phenotype into four stages. In the first stage (stage 1, S1), on 19 April 2016, the flag leaves of *m68* and the WT showed almost no differences (Figure 2A). In the second stage (stage 2, S2), on 25 April, the *m68* flag leaf tip exhibited senescence (Figure 2B). Then, the flag leaf of *m68* underwent accelerated senescence, and senescence was obvious on 3 May 2016 (stage 3, S3) (Figure 2C). In the final stage (stage 4, S4), the flag leaf of *m68* had almost completely turned yellow, while the flag leaf of the WT had not senesced (Figure 2D). Our measurements of maximum quantum efficiency of photosystem II (F_v_/F_m_) and the malondialdehyde (MDA) contents indicated that in *m68* leaves, F_v_/F_m_ gradually decreased (Figure 2E), but the MDA content increased significantly as the leaf senescence progressed (Figure 2F). The expression of *TaPAO*, which is associated with chlorophyll degradation, increased rapidly during leaf senescence in *m68* (Figure 2G).

### 2.2. RNA-Seq Analysis and DEGs

In total, 24 sample libraries were constructed and sequenced. We obtained 40.79 to 41.51 million reads from each sample. The GC content of the raw reads ranged from 53.66 to 56.33% in different libraries, and the Q30 percentage exceeded 94.5%. These libraries contained 85.61–89.34% of mapped reads and 77.81–82.53% of unique reads mapped to the *T. aestivum* _CS42_TGAC_v1 (Table 2).

**Figure 3:**
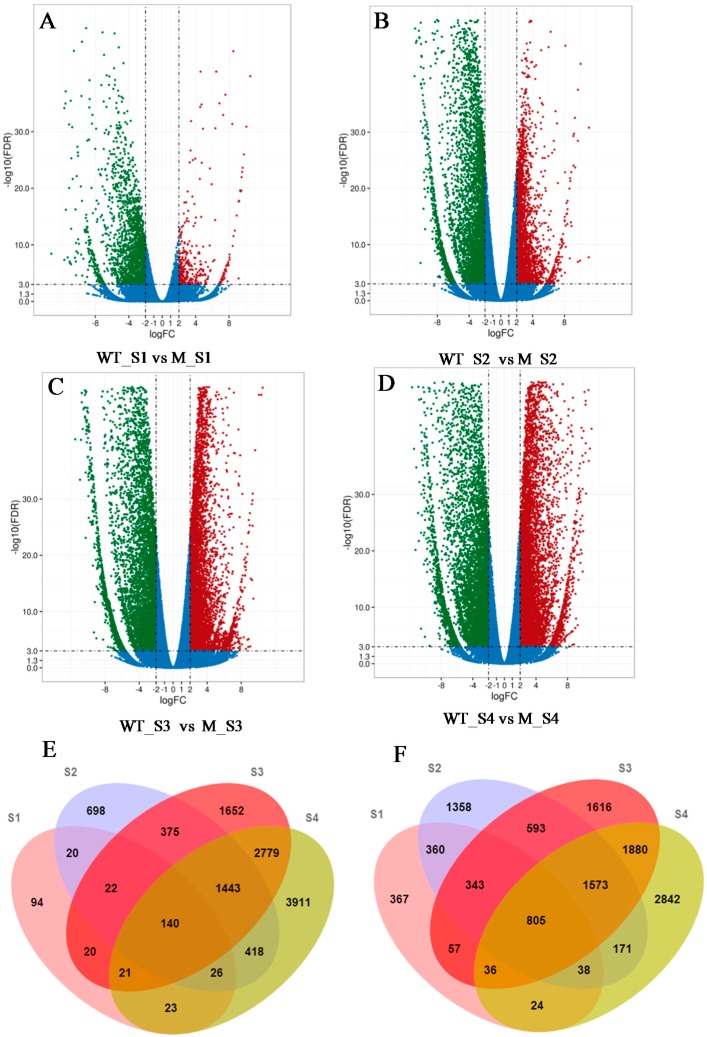
Volcano plots and Venn diagrams of DEGs. (**A**–**D**) DEGs between the WT and *m68* at different stages shown by volcano plots. The *X*-axis represents the fold change difference in the expression of genes in different comparison groups, and the *Y*-axis indicates the adjusted *p*-values for the differences in expression. The green dots represent up-regulated genes in *m68*, and the red dots represent down-regulated genes in *m68*. Blue dots indicate genes without significant changes in expression; (**E**) Venn diagrams indicate the overlap in down-regulated genes between *m68* and WT at different leaf senescence stages; (**F**) Venn diagrams indicate the overlap in up-regulated genes between *m68* and WT at different leaf senescence stages. The numbers in each circle represents the down-regulated or up-regulated genes at different leaf senescence stage.

The differentially expressed genes (DEGs) between *m68* and the WT at the four development stages were identified to investigate the genes that might be responsible for leaf senescence in the *m68* mutant (Supplementary Table S1). At S1 (WT_S1 vs. M_S1), although the flag leaf of the *m68* mutant had not started senescing, 2396 genes were significantly differentiated. These included 2030 up-regulated and 366 down-regulated genes in *m68* (Figure 3A). When the *m68* flag leaf tip exhibited senescence at S2, 8383 DEGs were identified (WT_S2 vs. M_S2), including 5241 up-regulated and 3142 down-regulated genes in *m68* (Figure 3B). During S3, the leaf senescence phenotype of *m68* was obvious, and more DEGs between WT_S3 and M_S3 were found, including 6903 up-regulated and 6452 down-regulated genes in *m68* (Figure 3C). At S4, the *m68* mutant flag leaf had turned completely yellow. The number of DEGs between WT_S4 and M_S4 increased to 16130, which included 7369 up-regulated and 8761 down-regulated genes in *m68* (Figure 3D). Moreover, we also detected the specific DEGs at different stages (Figure 3E,F); the Venn diagrams showed that 94, 698, 1652, and 3911 genes were down-regulated at S1, S2, S3, and S4, respectively, while 367, 1358, 1616, and 2842 genes specific were up-regulated at S1, S2, S3, and S4, respectively. The numbers of consistently down- and up-regulated genes in all of the stages were 140 and 805, respectively.

### 2.3. GO Enrichment Analysis of DEGs

**Figure 4:**
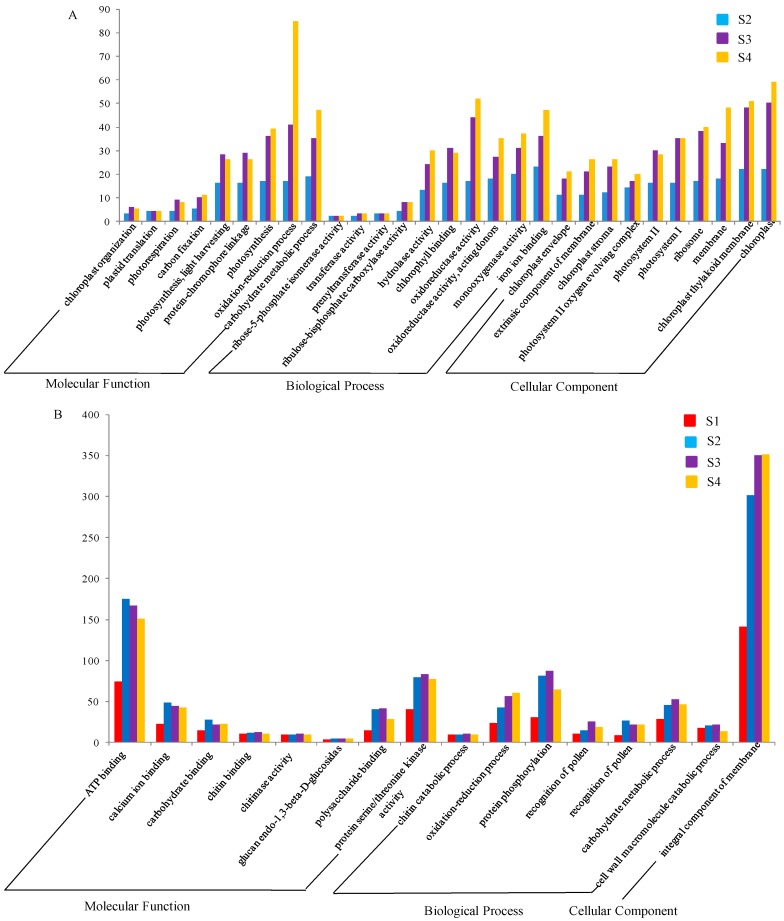
Gene Ontology (GO) enrichment analyses of down- and up-regulated DEGs between the WT and *m68* at different stages. Twenty-nine and sixteen GO terms are shown in down- and up-regulated DEGs, respectively. (**A**) GO enrichment analyses of down-regulated DEGs; (**B**) GO enrichment analyses of up-regulated DEGs; The *Y*-axis indicates the number of genes belonging to the GO terms below. The *X*-axis indicates GO terms. The DEGs at S1, S2, S3, and S4 are shown in red, blue, purple and yellow, respectively.

The enriched GO terms of down- and up-regulated DEGs at different time points were analyzed (Figure 4 and Supplementary Table S2). A total of 141 down-regulated DEGs were significantly enriched in GO terms. Among these terms, none were found at S1 (Figure 4A). However, 52 were found at S2, such as the chloroplast thylakoid membrane, photosynthesis, light harvesting, photosystem II, and chlorophyll binding. Ninety-one terms, such as ribulose-bisphosphate carboxylase activity, fructose 1,6-bisphosphate 1-phosphatase activity, ribosome, carbohydrate metabolic process, and chloroplast, were detected at S3. At S4, there existed 108 GO terms, among which, 29 were simultaneously enriched at S2 and S3 (Figure 4A). In terms of the up-regulated DEGs, there were 143 significantly enriched GO terms (Figure 4B). At S1, there were 48 including thirteen stage-specific GO terms such as lyase activity phosphate ion transport, transcription factor activity, sequence-specific DNA binding and inorganic phosphate transmembrane transporter activity. Sixty-one terms, such as integral component of membrane, response to stress, sucrose alpha-glucosidase activity, chlorophyll catabolic process, and oxidation-reduction process, were detected at S2. Sixty and 80 were found at S3 and S4, respectively. Among the 143 significantly enriched GO terms, 16 were enriched at all four development stages (Figure 4B), including terms in calcium ion binding, carbohydrate metabolic process, oxidation-reduction process, and integral component of the membrane.

### 2.4. KEGG Pathway Analysis of DEGs

**Figure 5:**
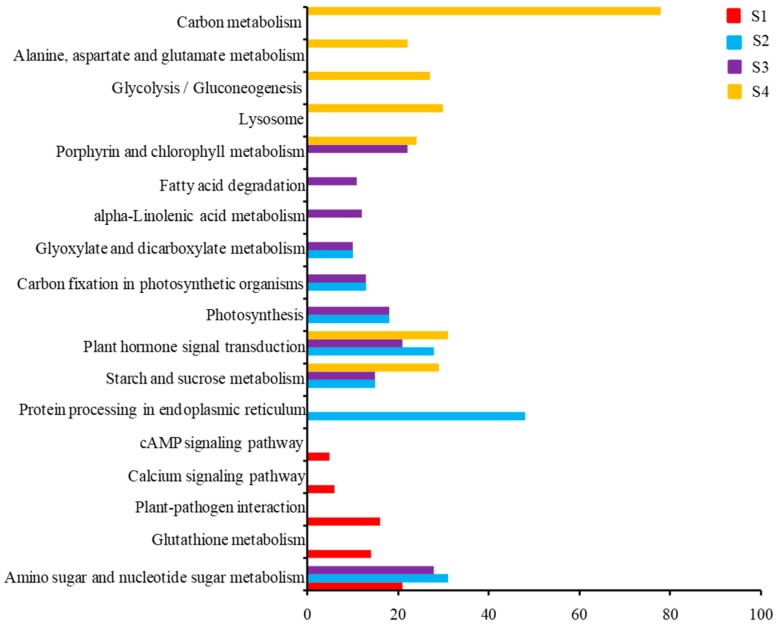
Kyoto Encyclopedia of Genes and Genomes (KEGG) analysis of DEGs between WT and *m68* in different leaf senescence stages. The *X*-axis indicates the number of genes belonging to the KEGG pathway on the left side. The DEGs in S1, S2, S3, and S4 are shown in red, blue, purple, and yellow, respectively.

To further investigate the leaf senescence pathway in the *m68* mutant, KEGG analysis of DEGs was performed at different stages. Among the four stages, DEGs were significantly enriched in 18 KEGG pathways (Figure 5). At S1, DEGs were enriched in 5 KEGG pathways, and four of them, i.e., the plant-pathogen interaction (ko04626), glutathione metabolism (ko00480), the calcium signaling pathway (ko04020), and the cyclic adenosine 3′,5′-monophosphate (cAMP) signaling pathway (ko04024), were only enriched in this stage. DEGs at S2 and S3 shared 6 enriched KEGG pathways, including starch and sucrose metabolism (ko00500), plant hormone signal transduction (ko04075), carbon fixation in photosynthetic organisms (ko00710), amino sugar and nucleotide sugar metabolism (ko00520), glyoxylate and dicarboxylate metabolism (ko00630), and photosynthesis (ko00195). At the late stage of leaf senescence, i.e., S4, DEGs were enriched in seven KEGG pathways, involving carbon metabolism (ko01200), lysosomes (ko04142), glycolysis (ko00010), plant hormone signal transduction (ko04075), starch and sucrose metabolism (ko00500), porphyrin and chlorophyll metabolism (ko00860) and alanine, aspartate, and glutamate metabolism (ko00250).

**Figure 6:**
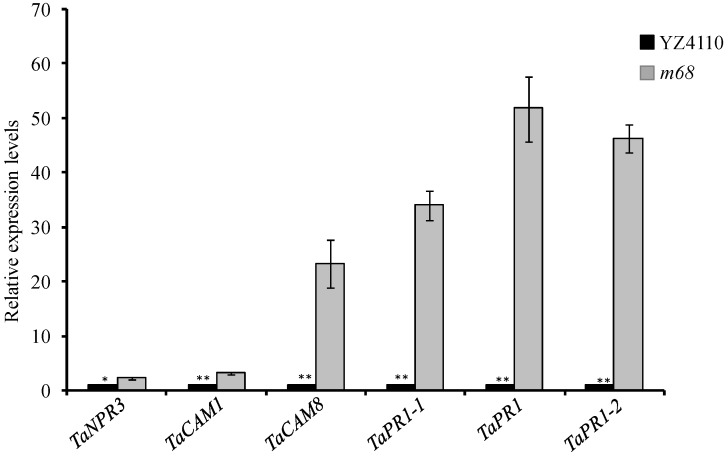
Salicylic acid (SA) signaling pathway-related gene expression levels analyzed using qRT-PCR. Each gene consisted of three biological replicates. Error bars represent the means ± SD (*n* = 3). *TaNPR3*, *TaCAM1*, *TaCAM8*, *TaPR1-1*, *TaPR* and *TaPR1-2* represent *MSTRG.18976.1*, *MSTRG.13794.1*, *MSTRG.27143.1*, *MSTRG.29737.1*, *MSTRG.32097.1*, and *MSTRG.26416.1*, respectively. *GAPDH* was selected as the internal standard. * indicates *p* < 0.05, ** indicates *p* < 0.01.

At S2, the *m68* flag leaf tips exhibited senescence, and many DEGs were significantly enriched in plant hormone signal transduction; therefore, genes that were involved in the plant hormone signal transduction pathway were selected and analyzed further. We identified 24 DEGs orthologs of the wheat genes related to ABA, IAA, ET, JA, and SA responses (Table 3). The orthologs of *IAA13*, *IAA7* and *IAA11*, *MSTRG.36133.1*, *MSTRG.30418.1*, and *MSTRG.37877.1* were down-regulated in the *m68* mutant. ABA signaling transduction-related gene expression levels increased in *m68.* In addition, the expression of several PR1 family-related genes, *MSTGR.29737.1*, *MSTGR.32097.1*, and *MSTGR.26416.1*, was significantly increased in the *m68* mutant. The orthologs of *NPR3* (*MSTRG.18976.1*), *CAM1* (*MSTRG.13794.1*), and *CAM8* (*MSTRG.27143.1*), which were also up-regulated, belonged to the SA signaling pathway. We checked the expression of six SA response gene orthologs using qRT-PCR (Figure 6). The results showed that the expression of these genes was obviously increased, which is consistent with the transcriptome analysis.

### 2.5. Expression Pattern Clustering Analysis of DEGs

**Figure 7:**
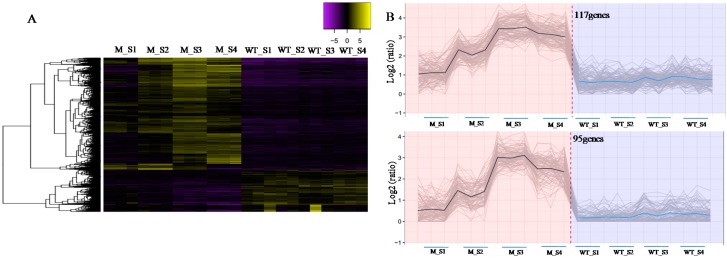
Clustering of DEGs between WT and *m68* at different leaf senescence stages. (**A**) Hierarchical clustering of all DEGs; (**B**) The two subclusters represent 27 subclusters which had increased expression pattern in *m68* and unchanged expression pattern in WT during leaf development. Gray lines show the relative expression levels of DEGs in the subcluster in WT and *m68* at different leaf senescence stages. Black lines and Blue lines show the average values for relative expression in each subcluster. *X*-axis indicates *m68* and WT at different leaf senescence stages. *Y*-axis indicates the relative expression level.

To identify genes closely that were related to senescence, we performed expression pattern cluster analysis of DEGs at different time points. All of the DEGs were used for cluster analysis. Hierarchical clustering based on DEGs (Figure 7A) and a 10% tree cutoff was performed using the R package cutree. In total, 4708 genes were classified into 393 clusters, which showed different expression patterns between the WT and *m68*. We further analyzed the genes of 27 subclusters, which contained 1012 genes (Figure 7B and Supplementary Table S3). The expression levels of these cluster genes were almost unchanged in the WT at all four time points, but in the *m68* mutant, they were obviously up-regulated during leaf senescence. There were 75 genes encoding kinase family proteins, 23 of which were receptor-like kinase genes. Additionally, 56 genes were annotated as transcription factors or DNA binding factors, 24 of which were orthologs of WRKY transcription factors, and 26 were orthologs of zinc finger transcription factors. In addition, 42 genes encoded different types of transporters, including inorganic phosphate transporters, high-affinity potassium transporters, boron transporter proteins, potassium transporters, ABC transporters, and amino acid transporters.

## 3. Discussion

In this study, we identified a wheat mutant, *m68*, which had many effects on plant development. Agronomic traits, such as grain weight, kernel number per spike, and floret number decreased significantly in *m68*. Because the leaves of *m68* started to senescence much earlier than those of the WT, we found some indicators that were detected in previous senescence studies. These senescence indicators include MDA, which is toxic to cells and can lead to cell death; F_v_/F_m_, which affects leaf photosynthesis and the accumulation of photosynthetic products; and, *TaPAO*, which participates in the degradation of chlorophyll [8,14]. We examined those indicators and found that the MDA content and the expression level of *TaPAO* were significantly up-regulated, and F_v_/F_m_ decreased remarkably in *m68* (Figure 2E–G). Therefore, *m68* is a premature senescence mutant. Using genetic analysis, we further determined that the premature senescence phenotype was controlled by a single recessive nuclear gene.

We randomly selected 16 genes among the DEGs to test their expression at different stages using qRT-PCR. Our qRT-PCR analysis results showed that those genes were indeed differentially expressed, which is in accordance with our transcriptome analysis results (Supplementary Figure S1). Therefore, our genome-wide transcriptome profiling analysis is reliable. The detection of specific DEGs at different stages indicated that the numbers of both the specific down-regulated and up-regulated genes were increased from S1 to S4. Through the GO enrichment analysis of down- and up-regulated DEGs at different time points, we detected a total of 141 and 143 enriched GO terms in down- and up-regulated DEGs at different time points, respectively. We found that the down-regulated DEGs did not have significantly enriched GO term at S1, while for the up-regulated DEGs, significantly enriched GO terms appeared at S1. For up-regulated DEGs, thirteen of 143 GO terms were specifically enriched at S1, which may play important roles in induced leaf senescence or onset of leaf senescence-related signal transduction, such as transcription factor activity and sequence-specific DNA binding. *AtWRKY53*, *AtWRKY70*, and *AtWRKY40* transcription factors participated in SA signaling transduction pathways [30,31,56], the expression levels of their orthologous genes were up-regulated in *m68*. *AtWRKY67* might participate in stress resistance-induced leaf senescence [57], and the expression levels of its orthologous genes were also up-regulated in *m68*. Therefore, the functions of these *WRKY* transcription factors might be very conserved in regulated leaf senescence and may include functions as upstream regulators of senescence. The mutant gene in *m68* might function upstream of the senescence regulatory network.

We also found that down-regulated DEGs were enriched in GO terms such as chlorophyll binding, chloroplast, chloroplast organization, photosystem I, and photosystem II at S2, S3, and S4, which were defined as the degenerative phase in previous studies [58]. In accordance with the results from previous studies, many chlorophyll metabolism genes were differentially expressed in *m68*, which might play important roles in wheat leaf senescence. For example, the expression of orthologs of chloroplast precursor-relative and TPR (tetratricopeptide repeat) genes, such as MSTRG.35978.1, *MSTRG.34045.1*, and *MSTRG.3773.1*, were reduced in *m68*. Chlorophyll synthesis-related genes, such as orthologs of *At5G54190*, *At1G44446* and *At3G51820* genes, were reduced in *m68*. *GATA* transcription factors can influence leaf senescence by regulating the chlorophyll content [17,18,19,20]. In our study, the *GATA* zinc finger family genes *MSTRG.14332.1* and *MSTRG.41348.1* were down-regulated during leaf senescence, which agrees with the function of *GATA* transcription factors. PPR proteins can influence the chloroplast content by regulating chloroplast gene expression, such as *OspTAC2* in rice [59] and *PPR53* can also affect chloroplast gene expression and leaf development in maize [16]. In our study, PPR-like genes *MSTRG.37348.1* and *MSTRG.23240.1* were down-regulated in *m68*. The above mentioned genes might play crucial roles in leaf senescence in wheat at the S2, S3 and S4 stages.

KEGG pathway analysis showed that plant hormone signaling severely affected the leaf senescence process. A previous study showed that ET, SA, JA, and ABA promoted leaf senescence, while CK, GA and IAA were observed to delay the progression of leaf senescence [21]. In our study, we found that the orthologs of the SA receptor gene *NPR3* and its downstream genes, *CaMs* and *PR*-related genes, such as *MSTRG.13794.1*, *MSTRG.27143.1*, *MSTRG.29737.1*, *MSTRG.32097.1*, and *MSTRG.26416.1*, were up-regulated in the *m68* mutant (Table 3). The JA response gene *JAZ2* was also up-regulated (Table 3). The expression levels of ABA signal transduction pathway genes, such as *ABF1*, *ABF4,* and *HAB1* significantly increased in *m68* (Table 3). The IAA signaling transduction-related genes *IAA7, IAA13,* and *IAA11* were down-regulated in the *m68* mutant (Table 3)*.* However, ET receptor gene *EIN2* expression was down-regulated in the *m68* mutant, which is supposed to promote leaf senescence (Table 3). These results seem to imply that leaf senescence in wheat is regulated by a variety of hormones and might have a unique regulatory mechanism.

The clusters of the expression pattern of DEGs provided us with a good opportunity to identify SAGs in wheat. We mainly paid attention to the clusters whose expression pattern had no obvious change during WT leaf development but showed a rapid increase during the leaf development of the mutant. There were 27 subclusters that contained 1012 genes showing this expression pattern (Figure 7B and Supplementary Table S3). All of the genes in this cluster were induced significantly during senescence and might be essential to wheat leaf senescence. Among these genes, many types of receptor-like protein kinases, transporter family proteins and transcription factors were enriched. The number of WRKY family and zinc finger transcription factors was much higher than that of the other transcription factors in these subclusters. It is suggested that they may play much more important roles than the other transcription factors in the regulation of leaf senescence in wheat. In addition, we noticed that flag leaf senescence occurred after the heading date, which was much later than that in the lower leaves of *m68*. Therefore, we deduced that oriented signal transduction and substance transport occur in plants around the heading date and are important in leaf senescence. We identified receptor genes and transporter genes that might participate in signal transduction and substance transport during leaf senescence of wheat.

## 4. Materials and Methods

### 4.1. Plant Materials and Genetic Analysis

The *m68* mutant was generated from an EMS mutant library of the common wheat cultivar Yanzhan 4110 (YZ4110), which was constructed and conserved in our laboratory. We planted *m68* and the YZ4110 in 2013–2016 in Beijing, and investigated agronomic traits, such as plant height and spike length each year.

We obtained inheritance-stable homozygote *m68* mutant seeds and crossed them with the YZ4110 to generate a heterozygote F_1_ population. F_1_ seeds were produced from self-crossed F_1_ plants and were sown on 7 October 2015, in Beijing. In each row, 15 seeds were evenly planted along a length of 2 m. We observed the flag leaves of 318 F_2_ plants on 3 May 2016. The number of plants exhibiting premature senescence was counted. A chi-square (χ^2^) test was used to analyze the segregation ratios.

### 4.2. Plant Materials Used in RNA-Seq

The WT and *m68* mutant were sown on 7 October 2015, i.e., 30 rows each, in Beijing (China), and were grown under field conditions. In each row, 15 seeds were evenly planted along a length of 2 m. The flag leaves of the WT and *m68* mutant were collected from the heading stage (19 April 2016) and were continuously sampled every 6 days until 9 May 2016. In each of the four stages, leaves from 10 plants were pooled to produce three biological replicates. All of the samples were immediately frozen in liquid nitrogen and were stored at −80 °C in a refrigerator.

### 4.3. F_v_/F_m_ and MDA Content Measurement

The F_v_/F_m_ and the MDA content of flag leaves of the WT and *m68* were measured at different sampling times. F_v_/F_m_ was measured using a handheld fluorometer (FluorPen FP100, Photon Systems Instruments, Drasov, Czech Republic), according to the manufacturer’s instructions. At each time point, 20 plants were measured; an average of three measurements were collected per plant. The MDA content was measured by utilizing a 5% thiobarbituric acid reaction, according to previous study methods [60].

### 4.4. RNA Extraction and Library Construction

Total RNA was isolated using Trizol reagent (Promega, Madison, WI, USA) following the manufacturer’s instructions. RNA purity was tested using a NanoDrop 2000 (Thermo, Waltham, MA, USA), RNA concentration was measured using an Agilent 2100 Bioanalyzer, and RNA integrity was evaluated with an Agilent RNA6000 NanoKit according to the manufacturer’s protocol.

Each sample required approximately 4 to 8 μg of total RNA to construct the RNA-Seq libraries. The method consisted mainly of the following steps: (1) mRNA was first purified from total RNA using polyA magnetic bead enrichment. (2) Chemical fragmentation was conducted. (3) Random hexamer priming was used to convert mRNA into first single-strand cDNA and to synthesize second-strand cDNA. (4) Sequencing adaptors were added to cDNA fragments. Suitable fragments were selected based on the separation by agarose gel electrophoresis. (5) PCR amplification construction of RNA libraries was completed. In total, 24 RNA libraries were constructed, and the quality and quantity of each RNA library were assessed using Nanodrop ND-1000 spectroscopy and an Agilent 2100 Bioanalyzer according to the protocol guide.

### 4.5. Illumina Sequencing and Read Mapping

RNA-Seq was performed using an Illumina HiSeq 4000 sequencing platform by OnMath Technologies, Chengdu, Sichuan, China. The raw reads were cleaned by removing adapter and low-quality sequences. Mapping of 24 sample reads to wheat genome (*T.* *aestivum.* TGACv1) was conducted with Tophat (version 2.1.0, default parameters). The new transcripts were assembled with StringTie (version 1.3.3b, default parameters). New gtf assembling and merging for each sample were used known TGAC gtf (version: TGACv1).

All of the sequencing data in this study are stored at the Sequence Read Archive under BioProject ID PRJA431543.

### 4.6. Transcriptome Analysis

The Kallisto tool, version v0.43.0 (https://pachterlab.github.io/kallisto), was used to calculate gene expression levels [61]. We use the edgerR tool, version v3.12.1, (http://bioinf.wehi.edu.au/edgeR) to analyze DEGs [62]. DEGs were defined as genes with a false discovery rate (FDR) <0.001 and |Log_2_(fold-change)| > 2.0.

GO enrichment analysis of DEGs was performed using the Goseq R package. All of the significantly DEGs between compare groups (both known genes and new assembled genes) were blasted in Pfam database (version 31.0, March 2017) and extracted all gene’s GO id for enrichment. GOseq was applied for enrichment and topGO was used for plot directed acyclic graph base on significantly enrichment gene (*p* value < 0.05 and *q* value < 0.05) [63]. We used KOBAS software 2.0 to determine significant enrichment of DEGs in KEGG pathways (*p*-value < 0.05 and corrected *p*-value < 0.05) [64]. Hierarchical clustering based on DEGs and a 10% tree cutoff was performed using the R package cutree.

### 4.7. Quantitative Real-Time PCR

To validate the RNA-seq data, we randomly selected 16 genes in DEGs to test their expression at different stages using qRT-PCR, which were the same RNA samples used for RNA-seq. According to the sequences, primer version 5.0 was used to design gene-specific primers. The primer sequences are listed in Supplementary Table S4. qRT-PCR was performed using a Roche LightCycler 480 Real-Time System (Roche, Switzerland). The wheat gene *GAPDH* (NCBI accession: AF251217.1) was used as an internal control for normalization of expression. Each experiment was performed with three biological replicates. qRT-PCR was performed using a 15-µL reaction volume that consisted of 7.5 µL of SYBR Mix (Toyobo, Japan), 1 µL of cDNA, 2 µL of gene-specific primers (2 µmol·L^−1^), and 4.5 µL of ddH_2_O. The PCR program parameters were 95 °C for 1 min, followed by 40 cycles at 95 °C for 20 s, 60 °C for 20 s, and 72 °C for 40 s. The relative gene expression levels were evaluated according to a previous study [65].

## Figures and Tables

**Table 1:** Comparison of agronomically important traits between YZ4110 and *m68.*

Accession	Years	Location	No. ^#^	Plant Height	Spike Length	Effective Tiller Number	Spikelet Number	Grain Number per Spike	Floret Number	1000-Grain Weight (g)
YZ4110	2014	Beijing	30	68.9 ± 1.1	9.9 ± 0.1	13.2 ± 0.2	24.9 ± 0.1	60.6 ± 4.5	79.5 ± 11.5	49.4 ± 0.8
*m68*			30	61.4 ± 0.5 **	8.8 ± 0.9	7.3 ± 1.3 **	17.3 ± 0.8 **	27.8 ± 3.9 **	50.8 ± 6.8 **	15.3 ± 0.5 **
YZ4110	2015	Beijing	30	66.8 ± 0.4	9.1 ± 0.8	15.3 ± 4.9	22.8 ± 0.7	48.7 ± 4.1	69.2 ± 7.8	50.9 ± 0.9
*m68*			30	57.4 ± 0.7 **	8.9 ± 0.1	8.1 ± 0.7 **	20.2 ± 0.1 *	33.9 ± 0.1 **	59.7 ± 8.4 *	15.7 ± 0.5 **
YZ4110	2016	Beijing	30	70.5 ± 1.7	8.9 ± 0.4	16.7 ± 2.4	23.1 ± 0.4	49.8 ± 2.9	72.8 ± 3.8	48.0 ± 0.9
*m68*			30	57.8 ± 1.5 **	9.0 ± 0.4	6.2 ± 0.5 **	19.9 ± 0.5 *	37.3 ± 3.1 **	63.6 ± 8.5 *	15.7 ± 0.8 **

No. ^#^ indicates the number of plants analyzed; * and ** indicate *p* < 0.05 and *p* < 0.01, respectively, according to Student’s *t*-test.

**Table 2:** Descriptive statistics of the sequencing data of the eight samples.

Sample ID	No. of Total Reads (Mb)	Read Length (bp)	Data Size (G)	Q30 (%)	GC (%)	Mappable Reads (%)	Unique Mapped Reads (%)	Multiple Mapped Reads (%)
WT_S1	41.3	150	6.19	95.71	55.66	88.92	81.33	7.59
WT_S2	40.8	150	6.12	94.93	56.33	89.69	78.56	8.40
WT_S3	40.9	150	6.13	95.31	55.00	89.34	79.57	9.77
WT_S4	41.5	150	6.23	95.48	55.00	87.88	79.59	8.29
M_S1	41.4	150	6.21	94.65	56.00	85.72	77.81	7.91
M_S2	40.6	150	6.13	94.84	55.33	85.61	79.64	5.97
M_S3	41.5	150	6.22	95.85	54.33	87.89	81.69	6.20
M_S4	41.3	150	6.19	95.69	53.66	88.75	82.53	6.22

**Table 3:** Plant hormone-related DEGs.

Wheat Gene ID	Gene Name	*E*-Value	LogFC	Participate in Signal Transduction Pathway	Reference
*MSTRG.35870.1*	*ABF1*	4.00 × 10^−35^	−2.08	ABA signaling	[40]
*MSTRG.10319.1*	*OST1*	1.00 × 10^−142^	−2.19	ABA signaling	[35]
*MSTRG.11222.1*	*SNF1-like*	1.00 × 10^−142^	−2.11	ABA signaling	[41]
*MSTRG.14188.1*	*HAB1*	6.00 × 10^−63^	−3.61	ABA signaling	[42]
*MSTRG.33951.1*	*ABF4*	1.00 × 10^−35^	−2.66	ABA signaling	[43]
*MSTRG.18662.1*	*HAI3*	8.00 × 10^−63^	−4.07	ABA signaling	[44]
*MSTRG.36133.1*	*IAA13*	3.00 × 10^−39^	2.31	AUX signaling	[45]
*MSTRG.30418.1*	*IAA7*	4.00 × 10^−47^	2.89	AUX signaling	[46]
*MSTRG.11304.1*	*SAAUR3*	0	−2.53	AUX signaling	[47]
*MSTRG.37877.1*	*IAA11*	3.00 × 10^−15^	2.12	AUX signaling	[48]
*MSTRG.10089.1*	*SAUR38*	2.00 × 10^−22^	−5.79	AUX signaling	—
*MSTRG.7713.1*	*SAUR38*	4.00 × 10^−20^	−9.86	AUX signaling	—
*MSTRG.41779.1*	*SAUR71*	4.00 × 10^−13^	−3.23	AUX signaling	—
*MSTRG.3553.1*	*AHP4*	1.00 × 10^−45^	3.37	CK signaling	[49]
*MSTRG.40221.1*	*ARR12*	2.00 × 10^−93^	2.68	CK signaling	[50]
*MSTRG.21216.1*	*EIN2*	1.00 × 10^−180^	2.07	Ethylene signal	[18]
*MSTRG.31221.1*	*JAZ2*	4.00 × 10^−12^	−3.69	JA signaling	[51]
*MSTRG.38578.1*	*PR1-like*	1.00 × 10^−39^	−3.94	JA signaling	[52]
*MSTRG.18976.1*	*NPR3*	1.00 × 10^−106^	−1.23	SA signaling	[53]
*MSTRG.13794.1*	*CAM1*	2.00 × 10^−81^	−2.20	SA signaling	[54]
*MSTRG.27143.1*	*CAM8*	2.00 × 10^−54^	−4.39	SA signaling	[55]
*MSTRG.29737.1*	*PR1-like*	7.00 × 10^−39^	−8.88	SA signaling	—
*MSTRG.32097.1*	*PR1-like*	7.00 × 10^−37^	−7.75	SA signaling	—
*MSTRG.26416.1*	*PR1-like*	4.00 × 10^−39^	−5.92	SA signaling	—

## Supplementary Materials

Supplementary materials can be found at http://www.mdpi.com/1422-0067/19/3/782/s1.
